# Case report: Urbanized non-human primates as sentinels for human zoonotic diseases: a case of acute fatal toxoplasmosis in a free-ranging marmoset in coinfection with yellow fever virus

**DOI:** 10.3389/fpubh.2023.1236384

**Published:** 2023-08-21

**Authors:** Davi E. R. Sousa, Tais M. Wilson, Isabel L. Macêdo, Alessandro P. M. Romano, Daniel G. Ramos, Pedro H. O. Passos, Gabriela R. T. Costa, Vagner S. Fonseca, Maria Angélica M. M. Mares-Guia, Marta Giovanetti, Luiz Carlos Junior Alcantara, Ana Maria B. de Filippis, Giane R. Paludo, Cristiano B. Melo, Márcio B. Castro

**Affiliations:** ^1^Graduate Program in Animal Science, University of Brasília, Brasilia, Brazil; ^2^Veterinary Pathology Laboratory, University of Brasília, Brasília, Brazil; ^3^Technical Group of Arbovirus Surveillance, General Coordination of Communicable Diseases, Department of Communicable Disease Surveillance, Secretariat of Health Surveillance, Brazilian Ministry of Health, Brasilia, Brazil; ^4^Environmental Health Surveillance Directorate of the Federal District, Brasilia, Brazil; ^5^Organização Pan-Americana da Saúde/Organização Mundial da Saúde, Brasília, Brazil; ^6^Instituto Rene Rachou, Fundação Oswaldo Cruz, Belo Horizonte, Minas Gerais, Brazil; ^7^Laboratório de Arbovírus e Vírus Hemorrágicos (LARBOH), Instituto Osawldo Cruz, Fiocruz, Rio de Janeiro, Brazil; ^8^Sciences and Technologies for Sustainable Development and One Health, University of Campus Bio-Medico of Rome, Rome, Italy

**Keywords:** non-human primates, *toxoplasma gondii*, infectious diseases, surveillance, One Health, zoonosis

## Abstract

Free-ranging non-human primates (NHP) can live in anthropized areas or urban environments in close contact with human populations. This condition can enable the emergence and transmission of high-impact zoonotic pathogens. For the first time, we detected a coinfection of the yellow fever (YF) virus with *Toxoplasma gondii* in a free-ranging NHP in a highly urbanized area of a metropolis in Brazil. Specifically, we observed this coinfection in a black-tufted marmoset found dead and taken for a necropsy by the local health surveillance service. After conducting an epidemiological investigation, characterizing the pathological features, and performing molecular assays, we confirmed that the marmoset developed an acute fatal infection caused by *T. gondii* in coinfection with a new YF virus South American-1 sub-lineage. As a result, we have raised concerns about the public health implications of these findings and discussed the importance of diagnosis and surveillance of zoonotic agents in urbanized NHPs. As competent hosts of zoonotic diseases such as YF and environmental sentinels for toxoplasmosis, NHPs play a crucial role in the One Health framework to predict and prevent the emergence of dangerous human pathogens.

## Introduction

Zoonotic infections have plagued populations since ancient civilizations and have again shown their drastic effects on public health recently in the COVID-19 pandemic. The surveillance of zoonotic diseases has become essential in a globalized world, especially considering wild animals that live or are close to people in anthropic environments. The early detection of epizootic yellow fever (YF) in non-human primates (NHPs) is a successful example of surveillance for triggering health actions to prevent YF cases in humans (1).

NHPs are important sentinels for YF that acutely die from the disease and play an essential role in disease transmission. In the sylvatic cycle of YF, New World NHPs are infected by mosquitoes in forests and may die. These animals and mosquitoes may also disseminate the YF virus (YFV) to anthropogenic areas such as cities through waves of epizootic outbreaks reaching urban NHP populations, where they can live as our daily neighbors and be potential hosts for the YFV and other zoonotic agents (1–6). In some countries, well-adapted species of NHPs are commonly found in cities and often have close contact with humans (1, 7, 8). Additionally, the high susceptibility of some free-ranging NHPs species to develop fatal *Toxoplasma gondii* infections can be a concern for public health services due to people’s outcry caused by the unexplained death of many animals during outbreaks (7, 9, 10).

The surveillance of deaths in free-ranging NHPs is an essential part of Brazil’s National Program to Control Yellow Fever (NPCYF) and also provides opportunities for the surveillance of other zoonotic agents in NHPs that live near human populations. In this study, for the first time, we detected an acute fatal case of toxoplasmosis in coinfection with the YFV in a free-ranging marmoset in an urbanized area of a metropolis in Brazil.

## Case presentation

**Figure 1:**
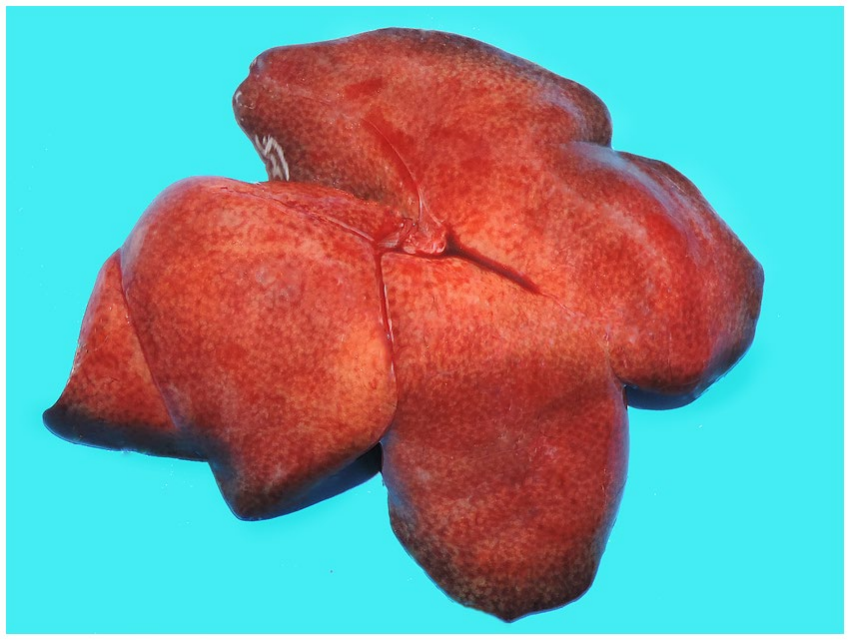
Gross hepatic changes in a case of acute fatal toxoplasmosis in coinfection with the YFV in an urbanized black-tufted marmoset (*Callithrix penicillata*). Hepatomegaly with an enhanced lobular pattern.

**Figure 2:**
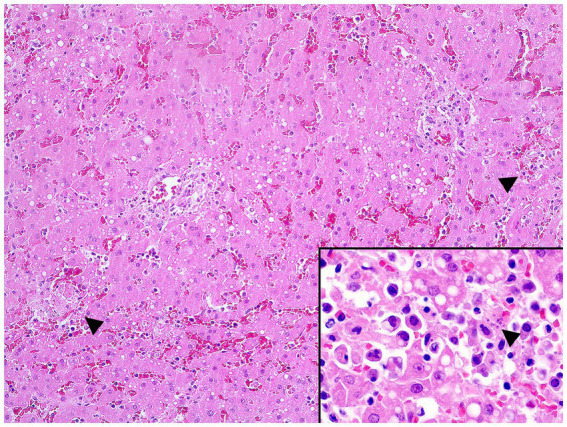
Multifocal random hepatocellular necrosis (arrowheads) (H&E, objective 10X) and free protozoal tachyzoites within a necrotic focus (arrowhead, inset) in the liver.

**Figure 3:**
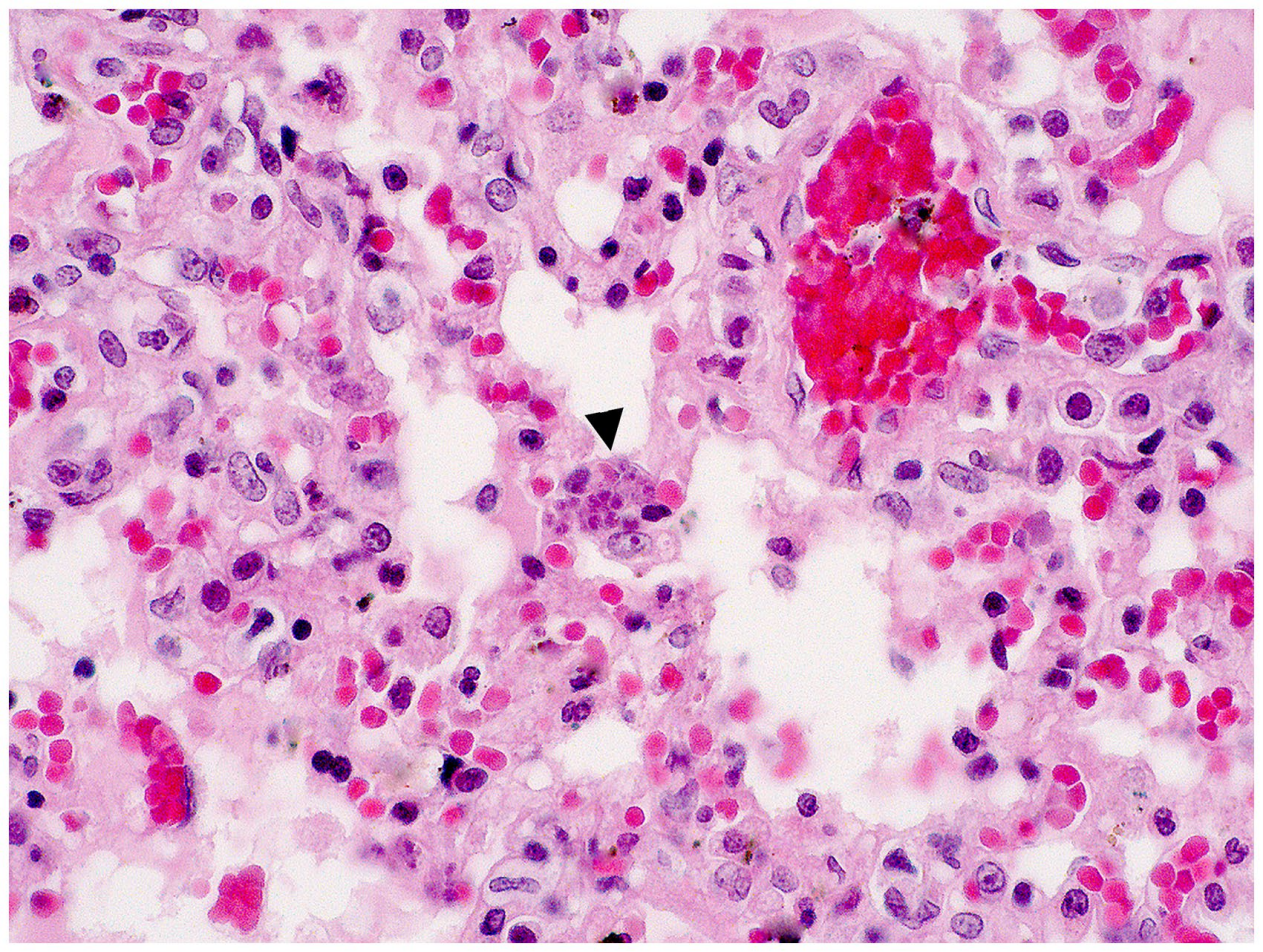
Interstitial pneumonia and intralesional protozoan tachyzoites (arrowhead) in the lung (H&E, objective 40X).

In December 2020, during a new re-emergence of YF in Midwestern Brazil, we investigated a black-tufted marmoset (*Callithrix penicillata*) found dead in an urbanized area of Brasilia (15°49′23.6”S 47°53′02.8”W), as part of the NPCYF at the Regional Reference Laboratory of the Brazilian Ministry of Health, University of Brasilia, Federal District, Midwestern Brazil. The marmoset carcass was fresh at necropsy and showed splenomegaly and hepatomegaly with an enhanced lobular pattern (Figure 1). Tissue samples were collected and stored at −20°C and also fixed in 10% neutral buffered formalin, paraffin-embedded, and stained with hematoxylin and eosin (H&E) stain for microscopic evaluation. Histologically, we observed multifocal random hepatocellular necrosis within scattered neutrophils and histiocytes and numerous free and intrahistiocytic intralesional protozoal tachyzoites (Figure 2). The lungs showed moderate interstitial pneumonia with bradyzoite cysts consistent with *T. gondii* (Figure 3), which were also similarly observed in the spleen.

We conducted immunohistochemical (IHC) assays for *T. gondii* and the YFV in paraffin-embedded tissue samples using anti-*T. gondii* polyclonal antibody (VMRD USA, dilution 1:2,000, incubated overnight) and anti-YFV monoclonal antibody (ACM3A8-C12 in-house produced by Fiocruz/PR, Brazilian Ministry of Health, dilution 1:200, incubated for 2 hours), and the alkaline phosphatase method (MACH 4 Universal AP-Polymer Kit, Biocare Medical), reaction revealed with a red chromogen (Warp Red Chromogen Kit, Biocare Medical) counterstained with Mayer’s hematoxylin. Antigen retrieval was performed with citrate pH 6.0 solution, 125°C, for 3 min in a pressure cooker (11).

**Figure 4:**
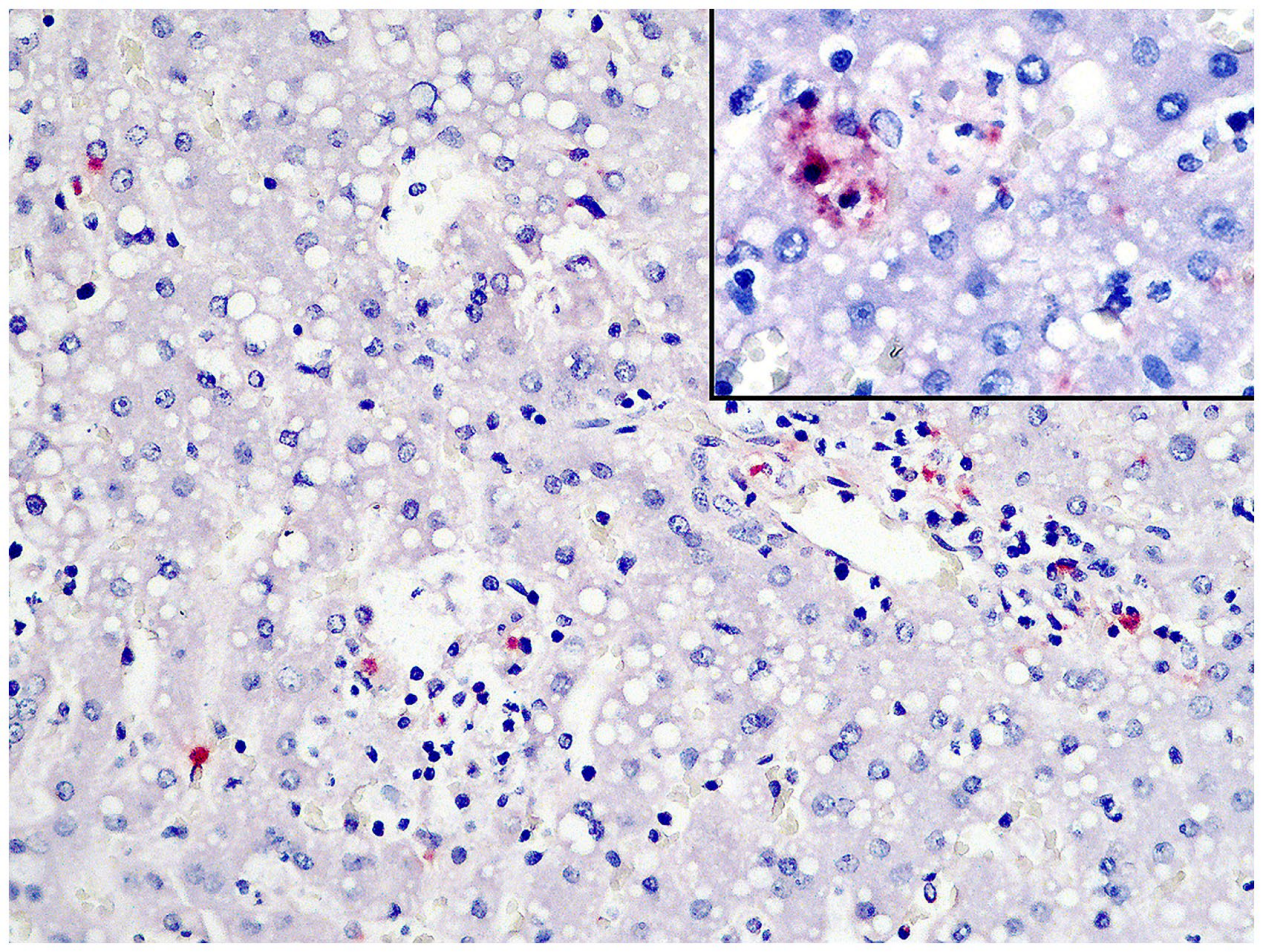
Free protozoan tachyzoites in the liver immunostained for *Toxoplasma gondii* within necrotic and inflammatory foci (IHC/AP, objective 20X) and in a close view (inset).

IHC revealed immunostaining of *T. gondii* tachyzoites and bradyzoite cysts in the liver (Figure 4), lungs, and spleen samples, while YFV antigens were not detected in the liver. We used conventional polymerase chain reaction (cPCR) in frozen liver samples to identify *T*. *gondii* DNA.

RNA was extracted from a tissue sample (liver) using the QIAamp Viral RNA Mini KitTM (Qiagen, Hilden, Germany) or the MagMAX Pathogen RNA/DNA Kit (Life Technologies, Carlsbad, United States) according to the manufacturer’s instructions. YFV RNA was detected using the RT-qPCR protocol previously described (12), with a cycle threshold value of 32.

The positive sample was submitted to a cDNA synthesis protocol (13) using a ProtoScript II First Strand cDNA Synthesis Kit. A multiplex tiling PCR was then performed using the previously published YFV primer scheme and 30 cycles of PCR using Q5 high-fidelity DNA polymerase (NEB) as previously described (14). Amplicons were purified using AMPure XP beads (Beckman Coulter), and cleaned-up PCR product concentrations were measured using a Qubit Double-stranded DNA (dsDNA) High Sensitivity (HS) Assay Kit on a Qubit 3.0 fluorometer (Thermo Fisher). DNA library preparation was performed using the Ligation Sequencing Kit (Oxford Nanopore Technologies) and the Native Barcoding Kit (NBD103; Oxford Nanopore Technologies, Oxford, United Kingdom). A sequencing library was generated from the barcoded products using the genomic DNA sequencing kit SQK-MAP007/SQK-LSK208 (Oxford Nanopore Technologies). The sequencing library was loaded onto a R9.4 flow cell (Oxford Nanopore Technologies).

Raw files were base called using Guppy v4.5.4, and barcode demultiplexing was performed using qcat. Consensus sequences were generated by *de novo* assembling using Genome Detective^1^ (15). The yellow fever typing tool (13) further revealed that the new genome sequence obtained in this study (GenBank accession number OP508570) represented a new sub-lineage belonging to the YFV South American-1 genotype (16) and was subsequently named YFV _PA/MG_ sub-lineage (17).

## Discussion

Considering the complexity of interactions between NHPs and human populations in urbanized environments, the detection of a fatal case of toxoplasmosis in coinfection with the YFV in a free-ranging marmoset highlights the importance of robust and broad laboratory-based diagnosis for infectious agents of public health concern in a wildlife animal as a sentinel for zoonosis. This case also emphasizes the need for constant public health surveillance services improvement, especially since both YF and toxoplasmosis can cause fatal outbreaks in marmosets (9, 10, 18) and less frequently in humans (19–21), and the geographical and clinicopathological features overlap.

The genomic surveillance analysis showed a new sub-lineage of the YFV in Central Brazil that initially emerged in the Amazon (Pará State, Brazil) and, for the first time, two distinct YFV sub-lineages have caused simultaneous outbreaks in non-human primates (NHPs) in very distinct regions and biomes of Brazil (6, 22). This new YFV _PA/MG_ sub-lineage identified in the Brazilian Cerrado (a savannah-like biome) has been spreading in a north-to-south axis from Amazonia (North Brazil) to other regions since 2017, which promoted fatal outbreaks in NHPs detected in 2020/2021 in Central Brazil (6, 17, 22). Concomitantly, a second sublineage named YFV _MG/SP/RS_ has been detected since 2018, causing epizootics in NHPs in Southern Brazil (22).

Epizootics of YF and toxoplasmosis are primarily characterized by severe liver damage in non-human primates, with some similarities in the gross aspect, but they have distinct histopathological features (18, 23). Midzonal to panlobular necrosis, Councilman body formation, and microvesicular steatosis are the main features of YF virus-induced liver damage (18). While in toxoplasmosis, mild to moderate random liver necrosis with a variable lymphohistiocytic inflammatory infiltrate intensity and intralesional apicomplexan zoites are the most significant pathological hallmarks (10, 23). In the urbanized marmoset, all pathological findings supported the diagnosis of acute fatal toxoplasmosis and no morphological changes indicated the YFV infection.

Surprisingly, the YFV coinfection molecularly confirmed in the black-tufted marmoset and with unremarkable hepatic virus-induced injury was an unexpected finding with great public health concern. Previous studies have shown that natural YFV infections were detected without causing viral liver damage and with a low viral load in marmosets found dead from other causes in urban and natural areas (4, 24). YFV infections occur in susceptible human and non-human primate populations in areas where mosquito vectors are present (18). *Haemagogus* spp. and *Sabethes* spp. mosquitos are the primary vectors for the sylvatic cycle of YF but have also been detected in periurban and forested areas of highly urbanized cities in Brazil (25, 26).

Regarding the urban zoonotic transmission of YF and this case of a YFV-infected marmoset, several factors must be taken into account: the presence of favorable conditions for the exposure of susceptible people; unvaccinated susceptible individuals; most marmosets usually have a low viral load, and their role in the maintenance and transmission of YF is not fully understood; and others (1, 4, 25, 27). Therefore, it is crucial to consider the risk of infected vector mosquitoes in the YFV transmission and maintenance in urbanized marmoset populations and also in the zoonotic viral spread for unvaccinated humans in natural parks and forested areas in the cities.

As observed in the marmoset of this study, New World NHPs are considered very susceptible to acute fatal toxoplasmosis, developing severe liver damage, interstitial pneumonia, and other systemic lesions in different organs (7, 10, 23). Severe toxoplasmosis is often associated with immunosuppression in humans, causing systemic lesions such as encephalitis, pneumonia, and hepatitis (28–30), but no such association has been documented in NHPs (31).

*T. gondii* is considered one of the most successful parasites worldwide, infecting around a third of the global population due to its ability to affect humans and a variety of warm-blooded animals, which is considered a pillar in successful protozoan dissemination and survival (32). Toxoplasmosis is also among the main foodborne parasitic diseases in the US and Europe (31, 32) and is a global public health concern, especially in developing countries (33, 34). In similar conditions observed in the marmoset, anthropized areas may play a critical role in the *T. gondii* spread from domestic cats to wildlife where they may come into contact (35).

*T. gondii* infections have mainly been reported in captive NHPs (7, 23) and with a few outbreaks in free-ranging marmosets (9, 10). Even though NHPs are not considered to be directly involved in the chain of disease transmission to humans (7), outbreaks of fatal toxoplasmosis in urbanized marmosets may serve as an indicator of potential environmental risk for *T. gondii* infection to people in a determined area or region.

Coinfections can significantly impact the transmission, clinical progression, and control of human infectious diseases, such as in the case of immunocompromised patients (such as those with AIDS) experiencing acute toxoplasmosis or being exposed to multiple other opportunistic infections. To date, coinfection of the YFV with other pathogens has not been reported in humans worldwide, but it is important to include it in the differential diagnosis of other icterohemorrhagic diseases such as acute leptospirosis (27).

## Conclusion

Assessing the risk of infection for human zoonotic pathogens, such as the YFV and *T. gondii,* in a metropolitan environment is challenging and still requires further research. NHPs can be both victims and reservoirs of zoonotic diseases such as YF and environmental sentinels for toxoplasmosis, playing a crucial role in the One Health framework. With robust complementary molecular methods, permanent epidemiological and pathological investigations of deaths in NHPs are essential for surveillance programs to prevent human cases of lethal zoonoses in vulnerable populations, particularly during YFV outbreaks. The role of NHPs as ecological sentinels for human toxoplasmosis is still overlooked, and further studies are needed to determine its relevance. Urbanized free-ranging NHPs living as our neighbors in backyards, gardens, and other forested areas in cities may serve as warning signs about the emergence of dangerous human pathogens.

## Data availability statement

The datasets presented in this study can be found in online repositories. The names of the repository/repositories and accession number(s) can be found at: https://www.ncbi.nlm.nih.gov/genbank/, OP508570.

## Ethics statement

This study was conducted on an animal found dead in the City, collected by the Health Surveillance Service and taken for a necropsy at the Official Regional Laboratory for Diagnosis of YF, Brazillian Ministry of Health. This Lab is part of the National Program for the Control and Prevention of YF.

## Author contributions

DS, TW, IM, and MC: data curation—original draft. AR, DR, PP, GC, VF, MM-G, MG, LA, AF, GP, and CM: writing—review. All authors contributed to the article and approved the submitted version.

## Funding

This work was supported in part by the National Institutes of Health USA grant U01 AI151698 for the United World Arbovirus Research Network (UWARN).

## Conflict of interest

The authors declare that the research was conducted in the absence of any commercial or financial relationships that could be construed as a potential conflict of interest.

## Publisher’s note

All claims expressed in this article are solely those of the authors and do not necessarily represent those of their affiliated organizations, or those of the publisher, the editors and the reviewers. Any product that may be evaluated in this article, or claim that may be made by its manufacturer, is not guaranteed or endorsed by the publisher.

## Footnotes

^1^https://www.genomedetective.com/
